# DISABKIDS^®^ in Brazil: advances and future perspectives for the production of scientific knowledge*

**DOI:** 10.1590/1518-8345.3003.3257

**Published:** 2020-04-17

**Authors:** Viviane Romeiro, Monika Bullinger, Maria Helena Palucci Marziale, Claudia Fegadolli, Roberta Alvarenga Reis, Renata Cristina de Campos Pereira Silveira, Moacyr Lobo da Costa-Júnior, Fátima Aparecida Emm Faleiros Sousa, Valéria Sousa de Andrade, Beatriz Juliana Conacci, Fernanda Karla Nascimento, Claudia Benedita dos Santos

**Affiliations:** 1 Universidade de São Paulo, Escola de Enfermagem de Ribeirão Preto, PAHO/WHO Collaborating Centre for Nursing Research Development, Ribeirão Preto, SP, Brazil.; 2 University Medical Center Hamburg-Eppendorf, Instituto de Medicina Psicológica, Hamburgo, HB, Germany.; 3 Universidade Federal de São Paulo, Instituto de Ciências Ambientais, Químicas e Farmacêuticas, São Paulo, SP, Brazil.; 4 Universidade Federal do Rio Grande do Sul, Faculdade de Odontologia, Porto Alegre, RS, Brazil.; 5 Scholarship holder at the Coordenação de Aperfeiçoamento de Pessoal de Nível Superior (CAPES), Brazil.; 6 Universidade Federal do Triângulo Mineiro, Departamento de Terapia Ocupacional, Uberaba, MG, Brazil.; 7 Scholarship holder at the Conselho Nacional de Desenvolvimento Científico e Tecnológico (CNPq), Grant # 311289/2017-7, Brazil.

**Keywords:** Cross-Cultural Comparison, Validation Studies as Topic, Surveys and Questionnaires, Psychometrics, Quality of Life, Review, Comparação Transcultural, Estudos de Validação como Assunto, Inquéritos e Questionários, Psicometria, Qualidade de Vida, Revisão, Comparación Transcultural, Estudios de Validación como Asunto, Encuestas y Cuestionarios, Psicometría, Calidad de Vida, Revisión

## Abstract

**Objective:**

to map the Brazilian scientific production related to the stages of the methodological process for the use of DISABKIDS^®^ instruments and/or forms adapted to Brazil.

**Method:**

scoping review, with searches conducted on10 electronic databases, plus Google Scholar and contacts with researchers, without restriction of period or language.

**Results:**

the mapping identified 90 scientific studies involving 46 instruments. Of these, 11 (23.9%) included the elaboration and/or cultural adaptation of the DISABKIDS^®^ instruments to measure the Quality of Life of children or adolescents with chronic conditions and 35 (76.1%) used the Generic Measures and/or Specific Modules for the semantic validation of other instruments.

**Conclusion:**

this scoping review allowed a comprehensive evaluation of the use of the DISABKIDS^®^ instrument and forms, in relation to the validation of the instrument adapted to Brazil, presenting a positive advance in the scenario with the development of academic/scientific projects in the country, incorporating the method recommended by the literature for the elaboration, cultural adaptation and validation of instruments and for the systematized and standardized recording of the perception and understanding of the target population about the measure of interest, using DISABKIDS^®^ forms adapted for this purpose.

## Abstract (pt)

**Objetivo:**

mapear a produção científica brasileira que versa sobre as fases do processo metodológico para a utilização dos instrumentos e/ou formulários DISABKIDS^®^ adaptados para o Brasil.

**Método:**

*scoping review* realizada através da busca em 10 bases de dados eletrônicas, no *Google Scholar* e por meio de consulta à pesquisadores, sem restrição de período ou idioma.

**Resultados:**

o mapeamento identificou 90 estudos científicos que envolveram 46 instrumentos, dos quais 11 (23,9%) foram realizados os processos de elaboração e/ou adaptação cultural dos instrumentos DISABKIDS^®^ para mensuração da Qualidade de Vida de crianças ou adolescentes com condições crônicas e em 35 (76,1%) foram utilizados o Formulários de Impressões Gerais e/ou Folhas Específicas para realizar a validação semântica de outros instrumentos.

**Conclusão:**

constatou-se que essa *scoping review* permitiu avaliação abrangente do uso do instrumento e dos documentos DISABKIDS^®^. Em relação à validação do instrumento adaptado ao Brasil apresenta avanço positivo do cenário junto ao desenvolvimento de projetos acadêmicos/científicos no país, a partir da incorporação ao método preconizado pela literatura para elaboração, adaptação cultural e validação de instrumentos, do registro sistematizado e padronizado da percepção, bem como do entendimento da população alvo sobre a medida de interesse, ao lançar mão de formulários DISABKIDS^®^ adaptados para esse fim.

## Abstract (es)

**Objetivo:**

mapear la producción científica que se ocupa de las etapas del proceso metodológico para el uso de los instrumentos y/o formularios adaptados DISABKIDS^®^ a Brasil.

**Método:**

*scoping review*, búsqueda realizada en 10 bases de datos electrónicas, en el Google Scholar y por intermedio de consulta a investigadores, sin restricción de período o idioma.

**Resultados:**

el mapeo identificó 90 estudios científicos que involucraron 46 instrumentos, de los cuales 11 (23,9%) la elaboración y/o adaptación cultural de los instrumentos DISABKIDS^®^ se realizaron para medir la Calidad de Vida de niños o adolescentes con afecciones crónicas y en 35 (76,1%) utilizaron los Formularios de Impresiones Generales y/o Hojas Específicas para realizar la validación semántica de otros instrumentos.

**Conclusión:**

se encontró que esta revisión de alcance permitió una evaluación exhaustiva del uso del instrumento y los documentos DISABKIDS^®^. En cuanto a la validación del instrumento adaptado a Brasil presenta un avance positivo del escenario en conjunto al desarrollo de proyectos académicos/científicos en el país, desde la incorporación al método recomendado por la literatura para la elaboración, adaptación cultural y validación de instrumentos, desde el registro sistematizado y estandarizado de la percepción, así como la comprensión de la población objetivo sobre la medida de interés mediante el uso de formularios DISABKIDS^®^ adaptados con esta finalidad.

## Introduction

The area of health constantly requires valid and reliable measures, with instruments that are calibrated to measure constructs applied according to standards. Instruments used for research and care assess mental, social and physical aspects and are aimed at achieving good health and making decisions about care and health policies(1-2).

It is important to develop instruments to measure morbidity or physical aspects; however, this process is less complex than the elaboration of instruments that evaluate constructs or characteristics related to human behavior. This fact has motivated the adaptation of previously constructed instruments that are appropriate to the socio-demographic or clinical specificities of the study population^(^ 3 ^-^ 5 ^)^.

In order to obtain reliable conclusions, studies that aim to measure subjective conditions with constructed or adapted instruments should have high methodological quality, both in the definition and in the measurement of the construct of interest^(^ 6 ^)^. They should provide clinically useful, meaningful and interpretable results, and psychometric properties such as validity, reliability and responsiveness should be assessed^(^ 1 ^,^ 7 ^-^ 8 ^)^.

In Brazil, the number of adaptations of instruments elaborated and validated in other cultures and the number of constructions of new questionnaires have been increasing. As a result, researchers have been doing this with the collaboration of international educational institutions and funding from government agencies^(^ 9 ^-^ 16 ^)^.

The project DISABKIDS^®^, from the European group DISABKIDS^®^, is a collaboration of seven European countries with the main objective of voicing the concerns of children and adolescents with chronic health conditions, as well as of their parents and caregivers. The project has constructed and refined tests of a system of instruments called DISABKIDS^®^ questionnaires^(^ 17 ^)^, translated into Brazilian Portuguese as *Instrumentos* DISABKIDS^®^. Among these, there are instruments that help the semantic validation process, referred in Brazilian Portuguese as *Formulário DISABKIDS^®^ de Impressão Geral* (DISABKIDS^®^ Chronic Generic Measure).and *Folha DISABKIDS^®^ Específica* (DISABKIDS^®^ Specific Modules).

DISABKIDS^®^ instruments are valid, reliable and sensitive, as well as fast to fill and easy to score and interpret^(^ 18 ^)^.

The objective of this study was to map the Brazilian scientific production related to the stages of the methodological process for the use of DISABKIDS^®^ instruments and/or forms adapted to Brazil.

## Method

The scoping review^(^ 19 ^)^ method, used in this study, has become popular in health research in recent years, as it does not restrict the parameters of the review to randomized controlled trials nor it requires quality evaluation of the studies included in the review^(^ 20 ^-^ 21 ^)^. The process is interactive and requires researchers to be involved in each step and, when necessary, to redo steps to ensure that the literature is comprehensively surveyed^(^ 19 ^,^ 21 ^)^.

According to the systematization proposed for scoping review studies, there are five mandatory stages and one optional stage: (1) identification of the research question; (2) identification of relevant studies; (3) selection of studies; (4) data mapping; (5) grouping, analysis and summary of data; and (6) contact with researchers (optional)^(^ 19 ^-^ 20 ^)^.

These stages guided this study, which also included considerations from other authors^(^ 21 ^-^ 22 ^)^. The research question must be designed to ensure comprehensiveness and depth^(^ 19 ^)^, and, in addition, it should be well structured, and contain information such as definition of concepts, target population, among others^(^ 21 ^)^. In addition, the question must be associated with the objective of the study^(^ 22 ^)^.

In the first stage, the research question was elaborated using the PICO strategy [acronym for patient (ou population), intervention, comparison, outcomes]. The use of this strategy directs the study and allows identifying keywords related to the theme. This helps the process of constructing the search strategy to find relevant studies in electronic databases, so that the best available scientific evidence can be located^(^ 23 ^)^. According to this strategy, P: DISABKIDS^®^ Instruments/Forms adapted for Brazil, I: the stages of the methodological process for the release of instruments, C: not applicable, as there are no comparisons in this study, and O: advances and perspectives of scientific knowledge in Brazil. Thus, the guiding question of this research was: “What are the advances and perspectives of scientific knowledge regarding the phases of the methodological process for releasing instruments according to the use of DISABKIDS^®^ instruments/forms adapted to Brazil?”.

To ensure the identification of relevant studies in the second stage, the search strategy occurred according to two processes. Initially, the researchers were consulted by two different means of communication.

The first one was Facebook, a free online public social network that is an important space for interaction and enables the sharing of questionnaires and transmission of information^(^ 24 ^)^. The Facebook profile called *“DISABKIDS no Brasil”* was used to invite all researchers to answer the research form, which had the objectives of collecting information regarding the use of DISABKDS^®^ instruments/forms in research and to provide access to these research for the collection of information.

The second mean was an e-mail sent to all professors, undergraduate and graduate students, and active nurses linked to a public Brazilian higher education institution. They were asked to respond to the research form.

Then, in December 2017, ten electronic databases were consulted: US National Library of Medicine National Institutes of Health (PUBMED), Cumulative Index to Nursing and Allied Health Literature (CINAHL), American Psychological Association (PsycINFO), Excerpta Medica dataBASE (EMBASE), Scopus, Web Of Science, Science Direct, Latin American and Caribbean Health Sciences Literature (LILACS), Brazilian Nursing Database (BDENF), Índice Bibliográfico Español en Ciencias de la Salud (IBECS) and the search engine Google Scholar (https://scholar.google.com.br/). Additionally, the bibliographic references of the studies included were contacted with specialists to check if there were any studies that were not included in the electronic search.

The search in the electronic databases did not limit period of publication nor language, and the only term used was “DISABKIDS”. Repeated studies were considered only once.

In the third stage, studies conducted in Brazil using DISABKIDS^®^ instruments/forms adapted to Brazil in the development of research, in part or in full, were included, regardless of the language of publication. Systematic or integrative reviews, opinion articles, comments, editorials or response letters were excluded.

**Figure 1:**
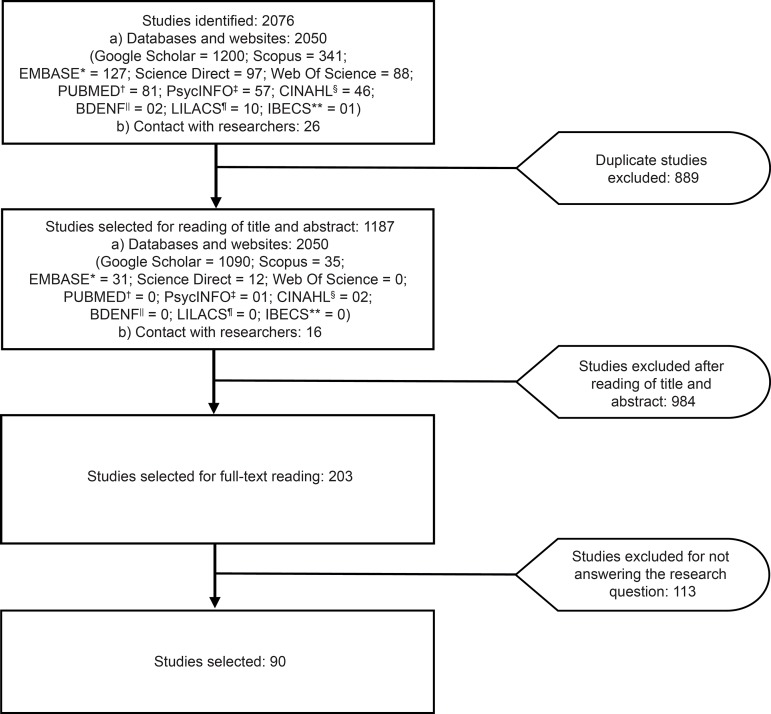
Flowchart of the study selection process - Ribeirão Preto, SP, Brazil, 2017 *EMBASE = Excerpta Medica dataBASE; ^†^PUBMED = US National Library of Medicine National Institutes of Health; ^‡^PsycINFO = American Psychological Association; ^§^CINAHL = Cumulative Index to Nursing and Allied Health Literature; ||BDENF = Brazilian Nursing Database; ^¶^LILACS = Latin American and Caribbean Health Sciences Literature; **IBECS = Índice Bibliográfico Español en Ciencias de la Salud

The studies were divided into two equal parts, and reviewed by two pairs independently. Each pair had a PhD researcher and all had expertise in methodological studies. The disagreements were settled by a third researcher, who was an associate professor, with vast and recognized experience in this process (Figure 1).

The fourth stage is mapping of relevant information for synthesis and interpretation of data. To answer the research question, data were extracted and mapped according to the variables: (1) ID (identification of the study); (2) title; (3) authors; (4) year of publication; (5) objectives; (6) population/participants (number of participants as reported in the document published by the author of each study included in the review), (7) chronic condition/functionality; (8) study type/design (as published by the study author); (9) study category (thesis, dissertation, article, annals, scientific initiation or final paper); and (10) means of publication.

To group and summarize the data collected according to the fifth stage, the following processes were identified: (1) elaboration of new instruments; (2) cultural adaptation of instruments; (3) validation of instrument (complete or pilot study); (4) application of the instruments; (5) use of the DISABKIDS^®^ structured questionnaire for focus groups; (6) use of the DISABKIDS^®^ structured questionnaire for focus group adapted for expert interviews; (7) use of the DISABKIDS^®^ Chronic Generic Measure; and (8) application of the DISABKIDS^®^ Specific Modules.

The studies were divided into two groups to identify the stages of the methodological process for the release of instruments: Group A, which included studies with elaboration and/or cultural adaptation processes of the DISABKIDS^®^ Instruments to measure Quality of Life of children or adolescents with chronic conditions; and Group B, in which the Generic Measure Forms and/or Specific Modules were used for the semantic validation of other instruments.

When verifying the release of instruments and the use of DISABKIDS^®^ forms, the stages were described according to groups of studies defined by “mother” projects, with the objective of avoiding duplicate information collection (Figure 2, Figure 3).

**Figure 2:** Distribution of studies belonging to Group A, according to the "mother" project, instrument and category. Ribeirão Preto, SP, Brazil

"Mother" Project	Instrument	Category
1	DISABKIDS^®^ Chronic Generic Measure	*Livre Docência* ^(^ 25 ^)^; thesis^(^ 26 ^)^; article^(^ 14 ^)^
2		Thesis^(^ 27 ^)^; congress^(^ 28 ^-^ 29 ^)^
3		Dissertation^(^ 30 ^)^; congress^(^ 31 ^)^
4	DISABKIDS^®^ Module - Living with Hearing Impairment	Thesis^(^ 32 ^)^
5	DISABKIDS^®^ - Cerebral Palsy	SI*∕Final paper^(^ 33 ^)^
6	DISABKIDS^®^ - Cystic Fibrosis Module	Dissertation^(^ 34 ^)^; congress^(^ 35 ^-^ 36 ^)^; article^(^ 11 ^)^
7		Thesis^(^ 37 ^)^; congress^(^ 38 ^)^; article^(^ 12 ^)^
8	DISABKIDS^®^ - Atopic Dermatitis Module	Dissertation^(^ 39 ^)^; article^(^ 13 ^,^ 40 ^)^
9		Thesis^(^ 41 ^)^
10	DISABKIDS^®^ - Asthma Module	SI*∕Final paper^(^ 42 ^)^
11		SI*∕Final paper^(^ 43 ^)^
12		SI*∕Final paper^(^ 44 ^)^
13	DISABKIDS^®^ - Arthritis Module	SI*∕Final paper^(^ 45 ^)^
14		SI*∕Final paper^(^ 46 ^)^
15	DISABKIDS^®^ - Epilepsy Module	SI*∕Final paper^(^ 47 ^)^
16		SI*∕Final paper^(^ 48 ^)^
17	DISABKIDS^®^ - Living with HIV^†^ Module	Congress^(^ 49 ^-^ 50 ^)^; thesis^(^ 51 ^)^
18		Dissertation^(^ 52 ^)^; congress^(^ 53 ^)^
19	DISABKIDS^®^ - Chronic Kidney Disease Module	Congress^(^ 54 ^-^ 55 ^)^; thesis^(^ 56 ^)^; article^(^ 9 ^-^ 10 ^)^
20	DISABKIDS^®^ - Obesity Module	SI*∕Final paper^†(^ 57 ^)^

* SI = Scientific Initiation;

† HIV = Human Immunodeficiency Virus

**Figure 3:** Distribution of studies belonging to Group B, according to the "mother" project, instrument and category. Ribeirão Preto, SP, Brazil, 2017

"Mother" project	Instrument	Category
01	Duke Anticoagulation Satisfaction Scale	Dissertation^(^ 58 ^)^
02	Adolescent Pediatric Pain Tool	Dissertation^(^ 59 ^)^
03	Cardiac Patients Learning Needs Inventory	Article^(^ 60 ^)^
04	Body Image Quality Of Life Inventory	Dissertation^(^ 61 ^)^; article^(^ 62 ^)^
05	Palliative Outcome Scale	Dissertation^(^ 63 ^)^
06	*Identificação da Prática de Enfermeiros nas Radiodermatites*	Dissertation^(^ 64 ^)^; article^(^ 65 ^-^ 66 ^)^
07	Appraisal of Self Care Agency Scale-Revised	Thesis^(^ 67 ^)^; article^(^ 68 ^)^
08	Patient Assessment of Chronic Illness Care	Thesis^(^ 69 ^)^
09	*Tecnologia educacional para a avaliação clínica de recém-nascidos prematuros*	Article^(^ 70 ^)^
10	Questionnaires for knowledge and Compliance with Standard Precaution	Article^(^ 71 ^)^; Thesis^(^ 72 ^)^
11	*Coordenação das redes de atenção à saúde pela Atenção Primária à Saúde*	Thesis^(^ 73 ^)^; article^(^ 74 ^-^ 75 ^)^
12	*Intervenção Educativa sobre a Medida Indireta da Pressão Arterial por profissionais de enfermagem*	Thesis^(^ 76 ^)^
13	Costs of caring for children with câncer	Article^(^ 77 ^)^
14		Dissertation^(^ 78 ^)^
15	Pain Assessment in Advanced Dementia	Dissertation^(^ 79 ^)^; article^(^ 80 ^)^
16	Comply with post-exposure management among health care workers	Thesis^(^ 81 ^)^; article^(^ 82 ^-^ 83 ^)^
17	United States Pharmacopeia Dispensing Information	Article^(^ 84 ^)^
18	*Mandala de avaliação*	Dissertation^(^ 85 ^)^
19	Food Choice Questionnaire	Article^(^ 86 ^)^
20	*Avaliação da Transferência do Tratamento diretamente observado*	Article^(^ 87 ^)^; Thesis^(^ 88 ^)^
21		Thesis^(^ 89 ^)^
22	Perceived Stimatization Questionnaire e Social Comfort Questionnaire	Article^(^ 90 ^)^; Thesis^(^ 91 ^)^
23	Tuberculosis Related Stigma	Thesis^(^ 92 ^)^; article^(^ 93 ^-^ 94 ^)^
24	Patient Activation Measure	Thesis^(^ 95 ^)^
25	Quality Of recovery - 40 item	Thesis^(^ 96 ^)^; article^(^ 97 ^)^
26	*Cartões da Qualidade da Dor*	Dissertation^(^ 98 ^)^
27	*Avaliação do impacto da capacitação dos Agentes Comunitários de Saúde em doenças sexualmente transmissíveis*	Thesis^(^ 99 ^)^
28	Genetic Counseling Outcome Scale	Dissertation^(^ 100 ^)^
29	Needs of Parents Questionnaire	Dissertation^(^ 101 ^)^
30	*Avaliação da necessidade de saúde de pessoas com deficiência física, auditiva e visual*	Thesis^(^ 102 ^)^
31	*Programa educativo sobre registro da pressão arterial em serviço hospitalar de emergência*	Thesis^(^ 103 ^)^
32	*Inventário de integração a vida universitária*	Thesis^(^ 104 ^)^
33	Diabetes Management Self-efficacy Scale	Article^(^ 15 ^)^
34	Test Oral Anticoagulation Knowledge	Article^(^ 16 ^)^
35	Functional Assessment of Chronic Illness Therapy-Spiritual Well-Being	Thesis^(^ 105)
36	*Questionário de conhecimentos sobre Práticas Forenses*	Dissertation^(^ 106 ^)^

To address the last stage of this review, aspects related to the means of dissemination of results, opportunities for knowledge sharing, and exchanges with those interested in the field studied should be included^(^ 20 ^)^.

In accordance with the resolution of the National Health Council, the project was approved by the Research Ethics Committee of the Ribeirão Preto College of Nursing, University of São Paulo (CAAE: 59431916.6.0000.5393). Before the beginning of data collection consisting of contact of researchers through an online questionnaire, the researchers received information about the project and had access to the Informed Consent Form (TCLE). They were informed that answering the questionnaire would imply signing the consent form.

## Results

Ninety scientific studies involving 46 different instruments that used DISABKIDS^®^ forms/instruments adapted to Brazil were mapped.

Among the 90 studies selected, 39 (43.3%) are directly related to DISABKIDS^®^ instruments that measure the quality of life of children and adolescents with chronic conditions - Group A (Figure 2); and the other 51 (56.7%) are studies that used DISABKIDS^®^ in the semantic validation stage of their research - Group B (Figure 3).

Most studies, 82 (91.1%), were conducted in the State of São Paulo, followed by three (3.3%) in Minas Gerais, two (2.2%) in the Federal District, one (1.1%) in Bahia, one in Rio Grande do Norte and one in Sergipe.

Among these studies, 29 (32.2%) are articles, 24 (26.7%) are thesis, 16 (17.8%) are dissertations, 11 (12.2%) are papers presented at scientific events, nine (10%) are undergraduate studies presented as final papers and one (1.1%) is a thesis of an Associate Professor.

A significant part (44.4%) of these studies were published in the format of scientific articles or in the annals of national or international conferences. Among the 29 articles published in scientific journals, 11 (37.9%) had international co-authorship, with at least one international author.

The impact factor of the journals ranged from 0.446 to 2.768. As for the Qualis CAPES (Higher Education Personnel Improvement Coordination) classification for Nursing, the publications are in journals with classifications A1 (5; 17.24%), A2 (13; 44.83%) and B1 (11; 37.93%).

In the stages of elaboration and cultural adaptation, there is a larger number of studies that opted for the process of cultural adaptation. Ten (17.9%) are “mother” projects, among the 56, related to the elaboration of instruments and 45 (80.4%) are related to the cultural adaptation stage (Table 1).

**Table 1:** Distribution of "mother" projects belonging to Groups A (n = 20) and B (n = 36) according to the stages of the methodological process for the release of instruments (n = 56) - Ribeirão Preto, SP, Brazil, 2017

Group		Elaboration	Cultural Adaptation	Validation	Application
		n (%)	n (%)	n (%)	n (%)
A	Yes	4 (7.1)	12 (21.4)	11 (19.6)	1 (1.8)
	No	16 (28.6)	8 (14.3)	9 (16.1)	19 (33.9)
B	Yes	6 (10.7)	33 (58.9)	20 (35.7)	5 (8.9)
	No	30 (53.6)	3 (5.4)	16 (28.6)	31 (55.4)

The validation process occurred in 31 (55.4%) studies, of which 14 (45.2%) performed the validation of the initial psychometric properties of the instrument studied and 17 (54.8%) were related to the conclusion of the validation process for Brazil - three of which concerned elaboration and the others concerned adaptation.

Among the studies found, 21 (37.5%) did not include any stage of the validation process, which may be directly associated with the increased understanding of the complexity of the process of validation of instruments for construct measurement. This fact occurs because these studies are inserted in the measurement theory. Therefore, all psychometric assumptions to support their validity and reliability should be verified before its use^(^ 6 ^-^ 8 ^,^ 107 ^).^

Considering the application of instruments elaborated, adapted and validated for Brazil, it was observed that they were applied in only one study in Group A and five studies in Group B.

Among the studies that used DISABKIDS^®^ forms adapted for Brazil, 39 (69.6%) corresponded to the Generic Measure and 44 (78.6%) to the Specific Modules. Group A included 11 (55%) and 12 (60%) projects, respectively. Group B, in turn, included 28 (77.8%) and 32 (88.9%) projects (Table 2).

**Table 2:** Distribution of "mother" projects belonging to Groups A (n = 20) and B (n = 36) according to the use of DISABKIDS Forms adapted for Brazil (n = 56). Ribeirão Preto, SP, Brazil, 2017

		DISABKIDS^®^ Structured Questionnaire for Focus Groups	DISABKIDS^®^ Structured Questionnaire for Focus Group adapted for expert interviews	DISABKIDS^®^ Chronic Generic Measure	DISABKIDS^®^ Specific Modules
		n (%)	n (%)	n (%)	n (%)
A	Yes	4 (7.1)	2 (3.6)	11 (19.6)	12 (21.4)
	No	16 (28.6)	18 (32.1)	9 (16.1)	8 (14.3)
B	Yes	0 (0.0)	0 (0.0)	28 (50.0)	32 (57.1)
	No	36 (64.3)	36 (64.3)	8 (14.3)	4 (7.1)

## Discussion

The results showed that ever since the introduction of the stages of elaboration, translation, cultural adaptation and validation of DISABKIDS^®^ instruments and forms in Brazil, 90 studies that presented at least one of the systematized methods were developed.

The studies were developed by researchers affiliated to recognized higher education institutions in Brazil and had products derived from scientific work, showing that the process addressed allows access to knowledge and training of researchers at different levels^(^ 108 ^)^ (Figure 1, Figure 2). The studies involved 46 different instruments that can be made available as valid and reliable measurement tools for use in various sectors in Brazil, such as health and education^(^ 12 ^,^ 67 ^,^ 71 ^)^.

Regarding the stages of elaboration and cultural adaptation, there is a larger number of studies that performed cultural adaptation, corroborating the recommendations of the scientific literature on these aspect^(^ 15 ^-^ 16 ^,^ 38 ^,^ 95 ^,^ 97 ^).^ In fact, the complexity and slowness of the process of elaborating an instrument for measuring subjective constructs have motivated the search and adaptation of previously constructed instruments^(^ 5 ^,^ 109 ^)^.

The process of inclusion of DISABKIDS^®^ adapted forms in the semantic validation stage shows that researchers are concerned not only with the translation of the items of an instrument, but also want these items to be relevant and comprehensible for the target population in the process of cultural adaptation^(^ 5 ^,^ 109 ^)^.

This result reinforces the understanding of the Brazilian scientific community, which advocates the use of a standardized method for the cultural adaptation of the items, giving voice to the participant, which contributes to the validity and reliability of the instrument, regardless of the culture^(^ 5 ^,^ 9 ^-^ 10 ^,^ 12 ^-^ 14 ^,^ 40 ^,^ 110 ^-^ 111 ^)^.

Giving voice to the participants has been a decision-making strategy in studies on Patient Reported Outcomes (PRO)^(^ 112 ^-^ 113 ^)^. In these studies, in addition to discussing the importance of the participation of patients for the quality of care, there is also concern with their literacy and with management strategies^(^ 14 ^,^ 109 ^)^.

The projects developed in Group B began two years after the implementation of the process for Brazil, with populations and contexts different from Group A, which included cardiac patients, coordination of health care networks, tuberculosis stigma, blood pressure, educational technology, among others. This indicates that the method was quickly incorporated and understood, and easily applied by the scientific community (Figure 3).

Attention to these details allows comparing, in different national and international scenarios, the impact of a condition and/or its management on people’s lives, in a standardized way, in multicenter tests or outcome evaluations^(^ 5 ^,^ 114 ^)^.

The application of these measures can help improving the quality of care provided to the general population. These measures related to planning can be driven by: *(i)* attributes measured individually, through instruments that assess certain processes, such as mental, physical and social aspects, and coping with various situations; or *(ii)* interventions tested through clinical trials or quasi-experimental studies, using scores derived from these instruments to compare results in different groups^(^ 9 ^,^ 83 ^,^ 115 ^)^.

Additionally, the use of these measures is relevant, as health is still strongly based on the biomedical model and focused mostly on the disease, and not on a biopsychosocial approach, which would incorporate health components at body and social levels, taking into account their functionality^(^ 3 ^)^.

As found in this scoping review, the fact that most studies were developed in the state of São Paulo may be associated with the lack of dissemination of the method to other educational institutions in the country. Another gap found refers to the scarcity of use of the instruments in clinical practice.

## Conclusion

This scoping review answers the guiding question of the research, as it presents a positive advance of the scenario of development of academic/scientific projects that include the method recommended by the literature for the elaboration, cultural adaptation and validation of instruments and for the systematic and standardized recording of the perception and understanding of the target population about the measure of interest, using DISABKIDS^®^ forms adapted for this purpose.

The results also show perspectives regarding the dissemination of the method throughout the country, which will allow the release of valid and reliable instruments that can be used in clinical practice, aiming at reaching a biopsychosocial approach, associated with improving the quality of health care provided to the population.

The results presented show a broad use of DISABKIDS^®^ instruments/forms adapted to Brazil, facilitating the complex and thorough process of adaptation or elaboration of instruments within the practice of researchers.
